# Juvenile Dermatomyositis: what comes next? Long-term outcomes in childhood myositis from a patient perspective

**DOI:** 10.1186/s12969-022-00754-y

**Published:** 2022-11-16

**Authors:** C. Boros, L. McCann, S. Simou, D. Cancemi, N. Ambrose, C. A. Pilkington, M. Cortina-Borja, L. R Wedderburn, on behalf of the JDM Cohort and Biomarker Study (JDCBS)

**Affiliations:** 1 University of Adelaide Discipline of Paediatrics Adelaide, Adelaide, Australia; 2 Paediatric Rheumatology, Alder Hey Children’s NHS Foundation Trust, Liverpool, UK; 3 Infection, Immunity and Inflammation Teaching and Research Department, UCL GOS Institute of Child Health, 30 Guilford Street, London, WC1N 1EH UK; 4 Blackrock Clinic, Blackrock, Co, Rock Road, Dublin, A94E4X7 Ireland; 5 Great Ormond Street Hospital London, London, UK; 6 Population, Policy and Practice Teaching and Research Department, UCL GOS Institute of Child Health, London, UK; 7 Great Ormond Street Hospital for Children (GOSH), NIHR Biomedical Research Centre, London, UK

**Keywords:** Juvenile, Myositis dermatomyositis, Outcome, Adolescent, Young adult

## Abstract

**Background:**

To describe long-term outcomes in JDM using patient questionnaires and link to longitudinal, prospectively collected data for each patient within the Juvenile Dermatomyositis Cohort and Biomarker Study, UK and Ireland (JDCBS) to determine outcome predictors.

**Methods:**

JDCBS participants aged ≥ 16y completed the SF36, HAQ and a questionnaire regarding current disease features, medications, education and employment. Data collected from the JDCBS included disease subtype, demographics, clinical and laboratory features. Intensity indices were calculated for physician VAS, modified skin DAS, CMAS and MMT8 by dividing area under the curve (AUC) from longitudinal score trajectories by duration of study follow-up (*y*). Relationships between questionnaire and JDCBS clinical / laboratory data were investigated fitting statistical models appropriate for cross sectional and longitudinal data.

**Results:**

Of 190 questionnaires sent, 84 (44%) were returned. Average age of respondents was 20.6 years (SD 3.9), time since diagnosis was 12.4 years (SD 5.0), age at onset was 9.2 years (SD 4.3), female to male ratio 4.25:1. Forty-nine (59%) self-reported persistently active disease, 54 (65%) were still taking immunosuppressive medication. 14/32 at school/higher education reported myositis adversely affecting academic results. 18–24 year-olds were twice as likely to be unemployed compared the UK population (OR = 0.456, 95% CI 0.24, 0.84, *p* = 0.001). Participants ≥ 18 years were three times as likely to be living with a parent/guardian (OR = 3.39, *p* < 0.001). SF36 MCS and MMT8 intensity index scores were significantly correlated (ρ = 0.328, *p* = 0.007).

**Conclusions:**

After 12.4 years, questionnaire responders reported self-perceived high rates of persistently active disease and medication use, reduced rates of employment and were more likely to live with a parent/guardian. Perceived persistently active muscle disease appeared to affect quality of life in these patients and was the most significant contributor to long-term outcomes. Our findings highlight the importance of including the patient perspective in the assessment of long term outcomes, so that that we can start to target initial management strategies more effectively based on a combination of clinical and patient-reported data.

**Supplementary Information:**

The online version contains supplementary material available at 10.1186/s12969-022-00754-y.

## Background

Childhood idiopathic inflammatory myopathies (IIM) are rare disorders, with an annual incidence of 1.9–4.0 million children per year [1]. Answering questions from parents and patients regarding long-term outcomes is difficult, not only because of the rarity of these conditions, but also because physicians often lose contact with patients when they transition from paediatric to adult services. Most previous studies investigating long-term outcomes in these patients have used cross-sectional clinical review with retrospective case-note data acquisition [2–4]. In addition, few have investigated patient-reported outcomes [5–7]. Knowledge of patient-reported long-term outcomes by studying a prospective patient cohort is important as it will facilitate refinement of current treatment algorithms and help provide better care in this patient group.

## Methods

The aims of this study were to describe long-term patient-reported outcomes in adolescents and young adults (≥ 16 years old) who had an IIM in childhood, in relation to education and employment opportunities, health-related quality of life, medication side effects and disease damage/ chronicity. In addition, we aimed to find potential outcome predictors using matched clinical and laboratory data contained in the Juvenile Dermatomyositis Cohort and Biomarker Study (JDCBS).

All patients were participants of the JDCBS [8], a prospective registry and repository commenced in 2000. At the time of this study, there were a total of 489 patients recruited to the JDCBS from 16 participating centres. Eight sites were able to participate due to local site restrictions, from which 190 young people were aged 16 years or older at the time of study and had diagnosis of IIM. Research ethics approval was obtained from the North East York Health Research Authority (reference MREC/1/3/2001). All participants provided written informed consent. The study was performed according to the declaration of Helsinki and good clinical practice guidelines. Clinical and laboratory data as well as biological specimens were collected as described [9]. Myositis specific antibodies were measured as described previously [10].

The Centre investigator and study coordinator of each site were provided with relevant study documents, timelines for recruitment and the password protected, de-identified registry identities of eligible participants at their site. Potential participants were provided with an introductory letter explaining the purposes of the study, a participant information sheet, a consent form and three questionnaires for completion. Two of the three were validated questionnaires (HAQ, SF36 [11]) and the third was a newly developed questionnaire to provide information regarding patient-perceived current and past disease features, medication, side effects, effects of myositis on growth and development, education and employment opportunities, as well as information regarding current living arrangements, fertility (females only), smoking habits and alcohol intake (Supplementary material: Figure S1 Patient questionnaire). The newly developed questionnaire was assessed for readability using the online SMOG (a simple measure of gobbledygook) readability formula and targeted to a reading age of 12 years [12]. It was reviewed by a JDM young person’s group for suitability and ease of completion prior to research ethics submission, with minimal changes recommended.

Questionnaires were sent to potential participants by mail with a reply-paid envelope provided, or completed in the outpatient clinic setting without assistance from family or clinic staff, and returned to the principal investigator for anonymised data entry.

Data collected from the JDCBS included, but were not limited to: sex, demographics, socio-economic status (as defined by post code quintiles at diagnosis and at the time of questionnaire completion), ethnicity, myositis subtype, date of diagnosis, date of symptom onset, clinical features, Childhood Myositis Assessment Scale (CMAS), Manual Muscle Testing-8 (MMT8), Physician Global Visual Analogue Score (VAS), modified skin Disease Activity Score (modified skin DAS [13], medications used and laboratory tests (muscle enzymes, inflammatory markers, autoimmune serology and myositis specific antibodies (MSA)).

### Statistical analysis

Relationships between questionnaire and JDCBS clinical / laboratory data were investigated fitting statistical models appropriate for cross sectional and longitudinal data.

#### Cross sectional analysis

Comparisons of proportions were analysed using Chi squared or Fisher’s exact test, and reported, where relevant, with odds ratios and 95% confidence intervals. To account for small sample sizes, in both tests, the null distribution was approximated using a bootstrap method with 10,000 replicates. Comparisons of continuous data were performed using Student’s *t*-test for normally distributed data or Wilcoxon-Mann–Whitney and Kruskal–Wallis tests for non-normally distributed data. Normality and homogeneity of variances were assessed using the Shapiro–Wilk and Bartlett’s tests. Correlation was assessed using Spearman’s correlation coefficient. ANOVA (relationships between Physical and Mental component scores on the SF36 and current disease features) and MANOVA models were fitted to compare continuous, normally distributed outcome variables adjusted by categorical covariates. Logistic regression models were fitted to compare binary outcomes adjusted by categorical and continuous covariates. Conditional quantile regression models were fitted to assess changes in median as a function of covariates [14]. Comparisons with the general population, as provided by the UK Office for National Statistics, (ONS), were performed using standardised rates [15].

#### Longitudinal analysis

**Fig. 1:**
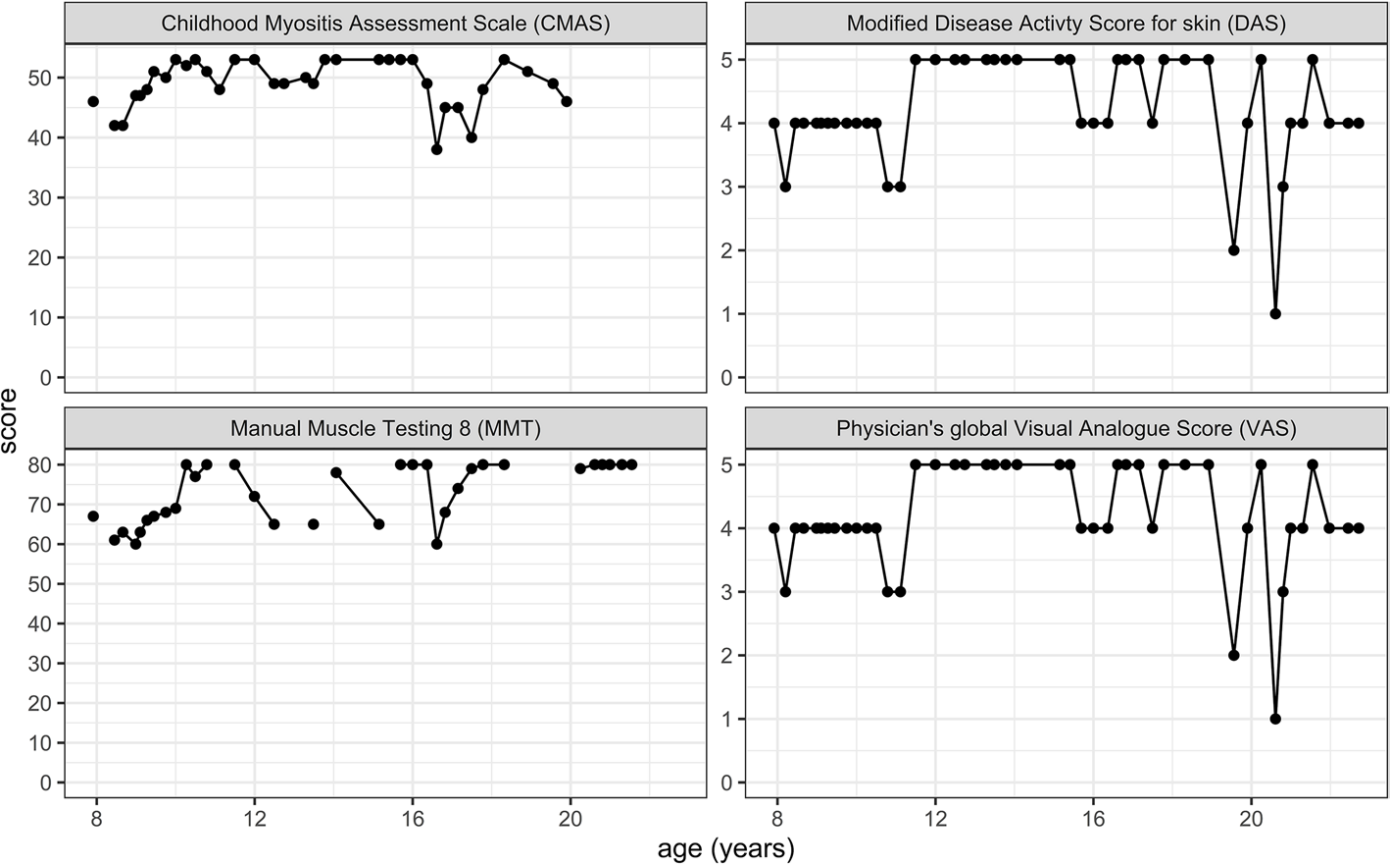
One individual longitudinal trajectory dataset. Data from one representative individual from visits over time are plotted for four variables; CMAS, modified skin DAS, MMT8 and Physician’s global VAS, showing variation in disease activity from age 8 years to 23 years of age

Longitudinal outcomes for each participant were summarised using trajectory analyses for CMAS, MMT, Physical global VAS and modified skin DAS [13, 16]. For each variable, the score for each visit documented in the JDCBS was plotted against time, to create an individual trajectory, as shown in Fig. 1. The area under the curve (AUC) for these trajectories was computed using the function areapl from the R library spatial [17] only for patients with at least three longitudinal observations.

Intensity indices for these variables were then calculated as the area under the curve (AUC) of individuals’ trajectories for each variable, divided by the duration of study follow-up in years. This index was computed only for individuals with data from at least three visits, and provided a summary of the disease activity for each variable during JDCBS follow-up. Since high MMT8 or CMAS indicate good muscle strength, in the case of these two measures a high index indicates better muscle strength or less weakness over time, than a low index. In contrast, a high modified skin DAS and high physician VAS indicate more disease burden.

All computations were performed in the R statistical language, version 3.5.2 [18]. A two-sided significance level of 0.05 was considered for all tests.

## Results

Of the 190 participants who were sent questionnaires, 84 sets of questionnaires (44.2%) were returned. Two sets of questionnaires were incomplete: one respondent failing to complete the newly developed questionnaire and another failing to complete the SF36. There were no significant differences between the two groups (responders and non-responders) and the relative proportions if IIM subtypes or for any of the other demographic data (Table 1). Both cohorts were predominantly white (69/84 (82.1%), and 84/106 (78.8%) respectively) with no significant differences in relative proportions of other ethnic groups. There were no differences in myositis specific antibody status at diagnosis or physician-assigned myositis sub-categories, between the two groups.

**Table 1:** Demographic data of questionnaire responders and non-responders

**DEMOGRAPHIC FEATURES**	**Study Cohort** ***n* = 84**	**Questionnaire Non-responders** (***n* = 106)**	***p*-value**
Current age, y, mean (SD)	20.6 (3.9)	22.3 (4.2)	NS
Oldest person, y	30.4	33.8	
F:M, n, ratio	*F* = 68, 4.25:1	*F* = 79, 2.9:1	NS
Age at onset, y, mean (SD)	9.2 (4.3)	9.0 (3.8)	NS
Disease duration, y, mean (SD)	12.4 (5.0)	14.0 (5.5)	NS

### Patient-reported outcomes

#### *Current disease activity (n* = *83 respondents)*

Among those who responded high rates of self-defined current disease activity were reported (Table 2): 49/83 (59%) felt that they still had active myositis, with 53 (64%) reporting at least one current disease feature. Twenty-one responders (25.3%) reported current skin rash and 18 of these 21 (85.7%) also reported calcinosis: these represent 21.7% of those who responded to the survey. Eight responders (9.6%) reported concurrent skin and muscle involvement. This is consistent with their own self-reported immunosuppressive medication use in 54 (65%), of whom 20 (24.1%) were taking two immunosuppressive medications and 8 (9.6%) taking three or more (Table 3). Although the most common medications were anti-malarials, *n* = 23 (27.7%) methotrexate, *n* = 22(26.5%) and prednisolone *n* = 10(12.0%). Ten patients (12.0%) reported they were taking biologic therapy. Analysis of data from the JDCBS at time of last available follow up in the study for the survey respondents indicated good disease control at last follow up, with median (IQR Q1-Q3) values of MMT8 = 80 (80–80); CMAS = 52 (51–52), Physicians VAS = 0.4 (0–1) and modified skin DAS = 0 (0–1).

**Table 2:** Patient-reported disease features

**Current disease features, self-reported and self-assessed**^a^ **(*n* = 83)**^b^	**Yes**	**No**	**Don’t know**
‘Still have myositis’	49 (59)	20 (24.1)	14 (26.4)
Arthritis	28 (32.5)	49 (59.0)	6 (7.2)
Muscle Weakness	28 (32.5)	51 (61.4)	4 (4.8)
Skin Rash	21 (25.3)	53 (63.9)	9 (10.8)
Calcinosis (*n* = 82)	18 (21.9)	60 (73.2)	4 (4.9)
Lipodystrophy (*n* = 82)	6 (7.3)	67 (81.7)	9 (11.0)
Gastrointestinal involvement	4 (4.8)	75 (90.4)	4 (4.8)
Pulmonary Hypertension	2 (2.4)	79 (95.2)	2 (2.4)
Lung Involvement	1 (1.2)	82 (98.8)	-

^a^None reported cardiac or neurologic involvement

^b^one respondent did not complete this questionnaire

**Table 3:** Patient–reported immunosuppressive medication use

**Current medications (*n* = 83)**	***n* (%)**
None	29 (35.0)
Anti-Malarial (Hydroxychloroquine or Chloroquine)	23 (27.7)
Methotrexate	22 (26.5)
Prednisolone	10 (12.0)
Azathioprine	8 (9.6)
Mycophenolate (Mofetil or Sodium)	7 (8.4)
Intravenous Immunoglobulin	6 (7.2)
Adalimumab	4 (4.8)
Infliximab	3 (3.6)
Rituximab	2 (2.4)
Tocilizumab	1(1.2)
Cyclophosphamide	0
Taking two medications^a^	20 (24.1)
Talking three or more medications^a^	8 (9.6)

^a^Patients on more than one medication are counted in more than one line of the table

Eighty-three percent (*n* = 69/83) reported that they were still under the care of a rheumatologist including 14 of these 69 (20.3%) who were no longer taking medication. None reported referral to an Oncologist as part of their long-term follow-up.

One participant reported requiring admission for medication side effects in the previous 12 months (Intravenous Immunoglobulin caused anaphylaxis). Twelve of 54 currently taking medication (22.2%) had stopped medication due to side effects in the last 12 months, with methotrexate side effects being most commonly reported as the cause (*n* = 5), followed by anti-malarials (*n* = 3), Vitamin D (*n* = 2), and IVIG (*n* = 2).

Supplementary Material, Table S1 shows disease features reported by questionnaire responders in terms of medication status. Those who were taking medication (*n* = 54) had significantly higher rates of reported active myositis and muscle weakness (*p* < 0.001 and *p* = 0.033 respectively) than those who were not taking medication (*n* = 29). There were no significant differences between the two groups for reported current rash, calcinosis, lipodystrophy, or arthritis.

#### Autoantibody status

Seventy-three of the 84 participants (88.1%) had Myositis-Specific Antibodies (MSA) and Myositis-Associated Antibodies (MAA) tested at diagnosis (Supplementary Material Table S2). Of these 73, 15 (20.5%) were negative and 11 (15.1%) had unknown bands. One patient was positive for both anti-NXP2 and anti-Mi2 MSA. Of the eight who were anti-Mi2 positive, five (62.5%) reported that they were still taking immunosuppressive medication. There was no statistically significant relationship between autoantibody status at diagnosis and myositis subtype medications used, or other questionnaire data.

#### Education and employment

Of the 83 who responded, 17 (20.5%) were still at school, 15 (18.1%) enrolled in higher education, 38(45.8%) employed and 13 (15.7%) unemployed. Forty-four per cent of those at school or enrolled in higher education (*n* = 14/32) reported that their academic results had been adversely affected by myositis: time missed due to myositis, muscle weakness and fatigue were all felt to be contributors to this. Two thirds of respondents in all groups found that myositis or its treatment had made it difficult to study.

Although a higher proportion of those who were unemployed (*n* = 3/13*,* 23.1%) had to retrain because of myositis than those who were employed (*n* = 2/38, 5.3%), the difference was not statistically significant. Fourteen of 50 (28%) respondents reported career compromise due to myositis: of these, 10 were employed and four were unemployed. Only 21/47 (44.7%) of 18–24 year-olds were employed. Overall our study participants were twice as likely to be unemployed compared to the corresponding age group in the UK population (OR 0.456, 95% CI 0.24, 0.84, *p* = 0.001: UK ONS).

#### Health-related quality of life

Physical component scores (SF 36 PCS) were higher in those who did not report self-perceived active myositis (*p* = 0.003), arthritis (*p* = 0.001) or muscle weakness (*p* = 0.0001). Mental component scores (MCS) were also higher in those who did not report arthritis (*p* = 0.03) or muscle weakness (*p* = 0.013). No statistically significant associations were found between SF36 MCS or PCS and current rash or calcinosis, disease duration, CMAS or MMT8 scores at time of diagnosis, employment status or socio-economic status.

#### Activities of Daily Living (ADLs)

HAQ scores in this cohort ranged from 0 to 1.75, (median 0, interquartile range, IQR 0–0.13). Eleven of 83 respondents (13.3%) required assistance with ADLs or used aids or devices. Two respondents required a wheelchair for mobilisation and a further two were using crutches.

#### Living arrangements

The proportions of those still living with a parent/guardian compared with 2016 UK ONS data (*n* = 83 respondents) using direct standardisation as described above. Sixty-two (74.7%) reported still living with a parent/guardian. Of the remaining 21, 10 were living with a partner, five with friends or a flatmate, three were living alone and three did not detail their current living arrangements further. We obtained similar proportions for 16–17 year-olds compared with UK ONS population data, but expected and observed rates differed substantially for the 18–24 year-old group, and for the 25–30 year-old group. The expected number of people aged 16 to 30 living at home for the ONS population distribution, yielded a standardised rate of 62.9%, which is still less than in our cohort (74.7%). The odds ratio was 3.39 (*p* < 0.001) thus showing that in general those in the study group were over three times more likely to live at home than the general population. We also fitted a logistic regression model with interaction terms by group and age: there were no significant differences between ONS and this study among 16–17 year-olds (*p* = 0.45), but the differences in the two older age groups were very significant (*p* < 0.001) especially for the eldest age group.

#### Growth and development, smoking and alcohol intake

A proportion of respondents thought that myositis affected their height (25%), weight (31%) or puberty (75%) (Supplementary Material, Table S3). Body Mass Index percentiles were significantly lower at the time of questionnaire than at diagnosis, *p* = 0.0136 (Supplementary Material, Table S4). While this was initially surprising we believe this may have been affected by a small number of cases recruited to JDCBS early in the study who arrived at secondary or tertiary care already on steroids which would raise the median BMI. Reported smoking rates were less than documented in ONS statistics (Supplementary Material, Table S5), but alcohol intake was similar to the general UK population (Supplementary Material, Table S6).

### Longitudinal analysis

Associations between intensity indices for CMAS, MMT Physician Global VAS and modified skin DAS scores demonstrated a positive correlation between MMT8 and SF36 MCS (ρ = 0.328, *p* = 0.007). Thus, those with less weakness, and therefore better muscle strength, during their time in the JDCBS study, reported better mental health outcomes at the time of this survey. This association remained significant (*p* < 0.001) after fitting a conditional quantile regression model to predict changes in median MMT score as a function of MCS after adjusting for education/employment. There were no other significant associations found between these variables and questionnaire data.

**Fig. 2:**
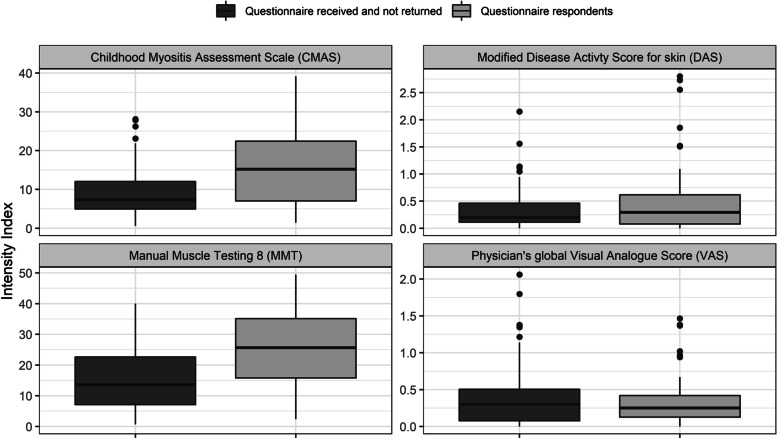
Intensity indices of disease activity data in questionnaire responders and questionnaire non- responders. Intensity index data for four variables, (CMAS, MMT8 modified skin DAS, and Physician’s global VAS as shown), comparing data for questionnaire responders and questionnaire non-responders as shown. Box plots show IQR values, line represents median value. Note that disease burden for MMT8 and CMAS indices is higher in those with a low index score; while for Physician’s global VAS and modified skin DAS a greater burden is indicated by a high index score

Given the availability of longitudinal clinical data available for the majority of subjects we next explored disease burden over time prior to this study on those who responded and those who did not. The number of questionnaire responders and non-responders with more than three variables for each of CMAS, MMT8, modified skin DAS and Physician Global VAS are shown in Supplementary material, Table S7. Figure 1 shows one patient’s longitudinal trajectories for these four variables over time. Figure 2 shows boxplots of the intensity indices (indicating an estimate of disease burden over time), for each variable. Questionnaire responders had significantly higher intensity indices than non-responders for CMAS (*p* = 0.004) and MMT (*p* < 0.001), indicating better muscle strength during follow-up within the JDCBS (since for MMT8 and CMAS high index value indicates good muscle strength), although there were no differences between these groups for modified skin DAS (*p* = 0.683) or Physician Global VAS (*p* = 0.994). However, we have no information regarding clinical outcomes for those who did not return the questionnaire, with which to analyse these data in greater detail.

## Discussion

This study is novel in that it has analysed data from an inception cohort of juvenile myositis patients followed prospectively from disease onset to investigate potential associations with long-term patient reported outcomes by linking longitudinal clinical and cross-sectional questionnaire data. Other larger studies have either not followed patients for the same duration or have not followed patients prospectively to collect long-term data [3, 19]. Other published long term outcome studies with similar or longer follow-up duration have all had smaller cohorts and concentrated mainly on disease damage/activity in cross sectional analysis with retrospective case-note data acquisition and with less focus on quality of life or other patient reported outcomes [2, 4, 20–25]. A key finding observed in this study is that patients with more weakness over their first 6–10 years of disease (measured by intensity indices for MMT8) were more likely to have a low SF36 score for their mental health subscale on follow up at the time of the study questionnaire. More work is needed, ideally with follow up of all cases to confirm this without bias and define clear predictors of patient reported outcomes.

Another strength of this study is the focus on the opinions and needs of adolescents and young adults who have had IIMs in childhood, in which there is also a paucity of published data. In those who responded to the questionaries, we found high self-perceived reported rates of ongoing muscle disease, skin disease, arthritis and need for immunosuppressive medication use in long term follow-up in those of our study cohort who responded to the survey, after a mean disease duration of 12.4 years. Self- perceived active muscle disease or arthritis appeared to affect quality of life outcomes in these patients and was associated with lower SF36 scores.

These data suggest that active muscle disease was perceived to be an important long-term outcome from a patient perspective in this cohort. Although not completely comparable due to different study design (cross-sectional retrospective cohort [2, 26, 27]) and data collected, other published studies have also found persisting disease activity and requirement of immunosuppressive medication use in long-term follow-up of childhood myositis patients. This may indicate that there are a higher number of patients with chronically active disease than previously thought, and that we need to develop strategies to identify patients at the onset of disease, who may need more intensive therapy in order to improve these outcomes. However, a clear limitation of our study is that there may have been differences between those who responded and those who did not. Thus, those who replied may have more ongoing active disease or negative outcomes than those who did not reply, which could inflate poor outcomes reported in this study.

In comparison with 3 other long term outcome studies reporting the HAQ (median 0, IQR 0–0.3 [26], median 0.4, IQR 0–1.0 [27], mean 0.2, SD 0.5, range 0–1.9 [2]), our cohort had better HAQ scores despite reports of persistently active disease.

This cohort also cohort had reduced rates of employment compared to the UK general population, in the 18–24 year old age group, and many felt that they had experienced career compromise or difficulties studying at school and/or in the tertiary setting as a result of their disease. This is similar to the cross-sectional case–control study by Tollisen et al. [6], in 39 adults who had juvenile onset Dermatomyositis, which found that despite similar educational attainment with controls and similar numbers of full-time workers in the two groups, patients with JDM had lower income than the controls. Five of 18 patients working part-time could no longer work full-time due to health problems.

Our study found no associations between socioeconomic status and any of the patient reported disease features or clinical variables. This is in contrast to a recently published study in which reported associations between low family income and worse scores of physical function, muscle strength, disease activity and quality of life scores in childhood IIM. However, this study followed patients only for a median of 3.1 years, much earlier in the disease course [19]. Our cohort was three times more likely to be living with a parent or guardian than the rest of the UK population, particularly for those over 24 years of age, according to comparative ONS data. This may well be related to the reduced employment rates and the resulting time taken to achieve financial independence.

Tollisen et al. also found reduced quality of life as measured by SF36 and the Health-Related Quality of Life (HRQOL) questionnaires. The total SF36 scores were significantly lower in the JDM patients than in the control population with correlations found between SF36 PCS and HAQ, the myositis damage index (MDI) and total disease activity score (DAS) [6]. Another published study from the same cohort (*n* = 48) used the SF36 to measure quality of life outcomes in long term follow-up of childhood myositis. The mean cross-sectional score for the SF36 was 51 (SD 9) similar to our cohort (mean 48.7, SD 9.4) and both close to average for norm-based scoring of this questionnaire, however Tollisen et al. did not report SF36 MCS [26]. Our study also documented SF36 MCS scores, which, as above, showed a significant correlation with MMT intensity index scores (ρ = 0.328, *p* = 0.007), indicating that for questionnaire responders, current quality of life from a mental health perspective, was directly related to the severity of reported muscle involvement. The impact of JDM on the mental health of young people and their families has been captured by recent studies [5, 28–30].

This important aspect was not addressed specifically at the time of this study. The SF-36 captured limited questions on the impact of emotional health on activities and energy. The bespoke questionnaire designed for this study asked if young people were under the care of other medical specialists and asked them to list medications, but did not specifically ask if they have sought access to counselling or psychology services, or if they had received treatment for anxiety or depression. We acknowledge that the presence of comorbidities such as depression or mental health issues may impact responses to the SF-36 and questionnaire.

It is now well recognised that inclusion of the patient perspective in healthcare is crucial not only to inform research priorities, but also to improve patient management and outcomes [31–33]. Patient-reported outcomes have been infrequently documented in children with inflammatory myopathies, particularly in long-term follow-up. Our study helps to address this gap, by combining long-term follow-up data with current patient perceptions, and has shown high reported rates of persistently active disease in this sub-group of adolescents and young adults and that this affects all facets of life.

There were also high reported rates of current medication use and muscle disease in questionnaire responders, thus indicating flare of or persisting muscle disease in longer-term follow-up. In explanation of this, we cannot exclude responder bias, and we cannot verify patient-reported outcomes; however, patient-physician discordance in disease activity is well documented not just for JDM, [34] but also in other paediatric [35] and adult rheumatic diseases [36]. In published studies, patient reports of disease activity are often higher than those of physicians. The discrepancy may be due to physician focus on objective measures of treatment response versus the patient focus on subjective experience of pain, disability or quality of life [36], which can lead to worse clinical and patient-reported outcomes. It would be useful to invite questionnaire responders for face-to-face clinical assessments to confirm their disease activity from a physician perspective.

Despite similar numbers of patients reporting active skin disease or muscle disease, with most of those who reported persisting rash also reporting concurrent calcinosis, there was no statistically significant difference in intensity indices for Phys VAS and modified skin DAS between those who did or did not return the questionnaire (Fig. 2). However, there were some missing values in the intensity index data, with significantly more questionnaire responders than non-responders having trajectories comprising at least three longitudinal values for the intensity indices for all four variables (Table S7). We did not carry out multiple imputation for these missing data, as we did not need to obtain standard errors of our model estimates.

There are several limitations of this study. An important limitation is that only 44% of those sent the questionnaires replied, and we have no disease medication or other long outcome term data on the other 46% of patients. Therefore, it is possible that our study over-estimated ongoing rates of active disease in patients with juvenile onset myositis. A further limitation is that we were not able to analyse for differences between those who filled in the questionnaires whilst they were in clinic versus those completing questionnaires at home since this metric depended on local logistics. We recognise that there may be differences between these groups that may further introduce bias, with those completing questionnaires in clinic more likely to be younger, still followed in the paediatric rheumatology clinic and possibly more likely to have ongoing active medical issues. Although we do not have specific data on this, we are aware that most sites sent questionnaires to patients by post and therefore most questionnaires would have been completed at home.

At the time of this study, specific questions on mental health status, such as if young people were suffering from depression or anxiety (either self-reported or diagnosed) were not addressed. Since this time, the impact of chronic disease on mental health status has increasingly been recognised, but this has not specifically been addressed in this study [5, 28–30]. We recognise that mental health comorbidities may influence responses given to questions in the SF-36 and study questionnaire.

A further limitation of this study is that the study questionnaire relying on perceived disease activity, which was not validated by a face-to-face consultation at that time. It is possible that perceived muscle weakness for example may be impacted by several factors including cardiorespiratory fitness, endurance, physical disability, or psychological wellbeing. However, the fact that over one-third of patients reported muscle weakness at the time of the questionnaire is comparable to other studies that demonstrate impaired muscle strength and cardiorespiratory fitness compared to healthy controls many years after the diagnosis of JDM [27, 37, 38].

## Conclusions

In conclusion this study suggests high levels of active disease, need for ongoing immunosuppressive medication and reduced quality of life in long-term follow-up of a proportion of children with IIM, despite good apparent initial disease control. Patient perception of currently active muscle disease appeared to be related to patient-reported lower health related quality of life in our cohort.

This cohort of patients also reported greater difficulties completing school and tertiary education, reduced levels of employment and a need to live with a parent or guardian for longer than the national average. These findings indicate that there may be substantially more long-term morbidity in a small but significant proportion of patients with childhood myositis than previously thought. Our findings also highlight the importance of including the patient perspective in the assessment of long term outcomes, to ensure not only that future research priorities can facilitate desired outcomes for patients but that we can also start to target initial management strategies more effectively based on a combination of clinical and patient-reported data.

## Supplementary Information

**Additional file 1:** **Figure S1.** Participant questionnaire. **Supplementary Table S1.** Patient reported disease features in questionnaire responders by medication status*. **Supplementary Table S2.** Myositis-Specific and Myositis-Associated antibody status of questionnaire responders. **Supplementary Table S3.** Responses to questions regarding growth and development. **Supplementary Table S4.** Height, weight and BMI percentiles at diagnosis and at time of questionnaire. **Supplementary Table S5.** Questionnaire data regarding smoking compared to UK ONS data. **Supplementary Table S6.** Alcohol intake of participants compared to UK ONS data. **Supplementary Table S7.** Numbers with ≥ 3 longitudinal values for CMAS, MMT8, modified skin DAS and Physician Global VAS.

## Data Availability

The datasets used and/or analysed during the current study are available from the corresponding author upon reasonable request.

## Abbreviations

JDM: Juvenile Dermatomyositis
JDCBS: Juvenile Dermatomyositis Cohort and Biomarker Study of the United Kingdom and Ireland
AUC: Area Under the Curve
Phys VAS: Physician Global Visual Analogue Score
Skin DAS: Skin Disease Activity Score
CMAS: Childhood Myositis Activity Score
MMT8: Manual Muscle Testing (8 groups)
SF36: Short Form 36
MCS: Short Form 36 Mental Component Score
PCS: Short Form 36 Physical Component score
HAQ: Health Assessment Questionnaire
SMOG: Simple Measure of Gobbledygook
ONS: United Kingdom Office for National Statistics
IIM: Idiopathic Inflammatory Myopathies
MSA: Myositis Specific Antibodies
MAA: Myositis Associated Antibodies
HRQoL: Health Related Quality of Life
MDI: Myositis Disability Index
