# Supplementary material for: Juvenile Dermatomyositis: what comes next? Long-term outcomes in childhood myositis from a patient perspective
Source: Pediatr Rheumatol Online J. 2022 Nov 16;20:102. doi: 10.1186/s12969-022-00754-y (PMC9670456; doi:10.1186/s12969-022-00754-y)
Supplement: Supplementary file 1 — Additional file 1: Figure S1. Participant questionnaire. Supplementary Table S1. Patient reported disease features in questionnaire responders by medication status*. Supplementary Table S2. Myositis-Specific and Myositis-Associated antibody status of questionnaire responders. Supplementary Table S3. Responses to questions regarding growth and development. Supplementary Table S4. Height, weight and BMI percentiles at diagnosis and at time of questionnaire. Supplementary Table S5. Questionnaire data regarding smoking compared to UK ONS data. Supplementary Table S6. Alcohol intake of participants compared to UK ONS data. Supplementary Table S7. Numbers with ≥ 3 longitudinal values for CMAS, MMT8, modified skin DAS and Physician Global VAS. [file 12969_2022_754_MOESM1_ESM.docx]

**Boros et al, submission 2022**

**Title:** Juvenile Dermatomyositis: What comes next? Long-term outcomes in childhood myositis from a patient perspective

**Additional material contains:**

**Figure S1 (pages 2-18)**

**and Tables S1-7**

**Supplementary material: Figure S1 participant questionnaire**

UK JDM Cohort and Biomarker study, 99RU11

Long term outcome form

First Name:

Gender: Male Female

Lastname:

Maiden name (if relevant)

Date this form filled in

**Section A General information about your myositis**

‘Myositis’ is the word used to describe redness and soreness of the muscles, also known as "inflammation of the muscles". There are two main types of myositis seen in young people. The most common is juvenile dermatomyositis (JDM) , where myositis is seen with a rash. Sometimes, young people have a type of myositis with no rash called juvenile polymyositis (JPM).

Don’t Know

No

Yes

Do you still have myositis?

No

Yes

Do you know what type of myositis you have/had?

If yes, please give type:

No

Are you still attending a Rheumatology Clinic?

Yes

Is this Paediatric Rheumatology?

No

Yes

Yes

No

Or Adult Rheumatology?

Would you give permission for us to contact your rheumatologist?

Yes

No

Hospital:

Name:

No

Are you seeing any other medical specialists (a doctor that is not your GP)?

Yes

Yes

No

If so, would you give permission for us to contact him/her?

If yes, please provide details

Name:

Hospital:

Are you on medication for your myositis?

If yes please tick which ones, if you know the names.

Don’t

Know

Yes

No

No

Yes

Have you ever been admitted to hospital because of medication side-effects?

**Section B. Current Medication/Medicines:**

If yes, when did this occur, what were the side effects and what treatment did you require?

Cyclophosphamide

Methotrexate

Prednisolone

Immunogloblin infusions

Plaquenil (hydroxychloroquine)

Other

Details of other medication

Have you stopped any of your medication due to side-effects in the past year?

Yes

No

If yes how many times and why?

Yes

Are any of these given by injection at home?

No

Yes

No

If yes, do you do your own injections?

Yes

No

Yes

No

Have you been admitted to hospital because of medication side-effects within the last year?

Have you ever stopped your medication due to side-effects?

?

If yes, when did this occur, what were the side effects and what treatment did you require?

If yes how many times and why?

No

Yes

If yes, when did this occur, what were the side effects and what treatment did you require?

Yes

No

Have you ever been to a hospital emergency department because of medication side-effects but not admitted to hospital?

In relation to your myositis, do you currently have any of the following?

**Section C. Your myositis at the moment:**

Yes

No

Don’t Know

Skin rash that you think is due to your myositis

Yes

No

Don’t Know

Muscle weakness

Don’t Know

No

Calcium under the skin (calcinosis)

Yes

Yes

No

Don’t Know

Loss of skin fat (lipodystrophy)

Yes

No

Don’t Know

Arthritis (pain in joints associated with swelling or stiffness)

Yes

No

Don’t Know

Gut disease

Don’t Know

No

Yes

Lung disease

Don’t Know

No

Yes

Heart disease

Don’t Know

No

Yes

High blood pressure in the lungs – called pulmonary hypertension

Yes

No

Don’t Know

Brain, spinal cord or nerve involvement

Have you ever had lung disease caused by your myositis?

Are you on any treatment for lung disease?

Don’t Know

No

Yes

Don’t Know

Yes

No

**Lung Disease**

**Section D.**

If yes, what treatment:

Have you ever any of the following investigations for lung disease caused by your myositis?

Lung function tests? Yes No Don’t Know

(tests of breathing and blowing)

Don’t Know

Yes

No

Chest Xray

?

Don’t Know

Yes

No

CT chest?

Yes

No

Don’t Know

Ultrasound (ECHO) to look for high blood pressure in the lungs?

(high blood pressure in the lungs is known as pulmonary hypertension)

Yes

No

Don’t Know

Exercise/Stress test

If you have answered yes to any of the above investigations, do you know the most recent results? Please provide any information you have

**Bones**

Don’t Know

Yes

No

Have you ever had a DEXA or bone density scan?

No

If yes, did this show that you had thin bones/osteoporosis?

Don’t Know

Yes

No

If yes, were you given any treatment?

If so what treatment?

Don’t Know

Yes

(Treatments might include calcium /vitamin D supplements or drugs called bisphosphonates)

If you have thin bones have you ever had a fracture

Don’t Know

No

Yes

caused by your thin bones?

**Skin**

Have you ever developed calcium (hard lumps) under the skin?

Yes

No

Don’t Know

due to your myositis ?

Yes

No

Don’t Know

Have you received treatment for this?

If yes, provide details

Yes

No

Don’t Know

Have you been diagnosed loss of skin fat (lipodystrophy )?

No

No

No

Yes

Yes

Yes

Don’t Know

Have you ever had high blood pressure?

**Heart**

No

Yes

If yes, do you need treatment?

Don’t Know

Don’t Know

Have you ever been told that you have high cholesterol?

If yes, do you need treatment?

Don’t Know

Have you had any of the following investigations for heart disease?

Yes

No

Don’t Know

Heart Ultrasound (ECHO) or ECG

Yes

No

Don’t Know

Exercise/Stress test

Yes

No

Don’t Know

Cardiac MRI

Yes

No

Don’t Know

Cardiac PET scan

If you answered yes to any of the above, do you know the most recent results? Please provide any information you have

**Gut/Bowels**

Have you had any of the following problems caused by your myositis?:

Stomach or bowel ulceration (ulceration is a sore in the gut associated with loss of the gut lining)

Yes

No

Don’t Know

Don’t Know

No

Yes

Bowel perforation (a hole in the gut)

Yes

No

Don’t Know

Gas in the bowel wall

Yes

No

Don’t Know

Chronic Constipation

Yes

No

Don’t Know

Slow stomach emptying

Yes

No

Don’t Know

Slow movement in the small or large bowel

Don’t Know

No

Yes

Pancreatitis (inflammation in the pancreas)

Yes

No

Don’t Know

Hepatitis (inflammation in the liver)

If you have replied “yes” to any of the above, please provide details of any relevant medical or surgical treatment you have had

**Growth and Development**

inches

cm

**.**

**OR**

What is your current height?

ft

**OR**

kg

ounces

**.**

What is your current weight?

lb

Has your myositis affected your height?

Compared to healthy people the same age, do you feel shorter?

Yes

No

Don’t Know

Yes

No

Has your myositis affected your weight?

Don’t Know

Don’t Know

Have you ever had treatment with growth hormones?

No

Yes

No

Do you think your myositis affected your puberty?

Yes

Don’t Know

If so, do you think you have caught up with healthy people the same age as you?

No

Yes

Don’t Know

**Q1. Education**

**Section D. Social and Educational Outcomes**

No

Are you still at school?

If no, please go to question 2.a.

If yes, which year are you in?

No

Yes

Yes

Do you plan to continue in further education?

If no, please go to question 2.b

If yes, details:

**If you are still in school and planning further education, please go to Section 2.c.**

How much school did you complete? Tick relevant box

**Q2.a. Qualifications**

- 7-10 years of primary school
- 1-2 years of high school
- 3 or more years of high school
- 1-4 years of college or university
- More than 4 years of college or university
- Other education

If other, can you please provide details

Do you think your school or university results were affected by your myositis?

No

Yes

Don’t Know

If yes,

Was this because of time missed in school/university?

Was it because of muscle weakness?

Was it because of fatigue / tiredness related to myositis?

Other reasons?

Details:

Don’t Know

No

Yes

Yes

No

Don’t Know

Yes

No

Don’t Know

Has your myositis or treatment ever made it difficult to study because you felt unwell?

No

Yes

Don’t Know

**Part time study**

If you are/were not in full-time school/university studies, can you please tick one of the following:

- I have previously been in full time study but cannot do so now because of my disease
- I have never been in full time study because of my disease
- I do/did not wish to study full time
- Other reasons ( please provide details )

**Q2.b. Employment**

Are you currently employed or have a job to go to?

Yes

No

Sick Leave

Full Time

Voluntary

Temporary

Part Time

Permanent

If yes, is the work (Tick all that apply):

If you are working part time, how many hours per week do you work?

What is your current salary?

If you aren’t currently employed, is this because of your myositis?

Don’t Know

Yes

No

Yes

Don’t Know

No

Have you had to compromise (change) your career choices because of your condition?

Have you ever had to retrain (for another job) due to your disease?

Yes

No

Don’t Know

Yes

No

Are you receiving social security payments?

No

Yes

Are you in a rehabilitation programme?

Yes

No

Are you receiving a pension?

Yes

No

Are you receiving disability benefits?

Are you unemployed because you prefer not to work?

Yes

No

**2.c. Driving**

No

No

Do you drive a car?

Do you have a drivers licence?

Yes

Yes

No

Yes

Have you ever smoked?

**2.d. Smoking**

Do you currently smoke? Yes No

If yes, age first smoked?

If so, how many cigarettes per day (average)

If e-cigarettes, how many per day (on average)

**2.e Drinking**

Have you ever drunk alcohol? Yes No

No

Yes

Do you currently drink alcohol?

If yes, number of alcohol units per week on average?

**Section F. Home**

Who do you live with?

Mum/Dad/Guardian

On your Own

With friends/flatmate

Partner

Other

No

Yes

Have you ever had a miscarriage?

No

Yes

Have you ever been pregnant?

Do you use birth control pills or injections?

No

Yes

**Section G. If Female**

**More Information**

If we need more information, are you happy to be contacted by the study team?

No

Yes

No

Yes

If yes, would you prefer to be contacted by: **Telephone**

If yes, best number for telephone contact

Best time(s) for contact

**Email**

No

Yes

Email address

If you feel happy to do so could you please provide your full date of birth and the post code of your current address:

These details will help us link the survey information to our study database

Date of Birth:

**Y Y**

**Y Y**

**MM**

**DD**

Postcode of your current address

**Supplementary Tables**

**Supplementary Table S1: Patient reported disease features in questionnaire responders by medication status***

|  | **Still have**  **Myositis** | **Current muscle**  **Weakness** | **Current skin**  **Rash** | **Current**  **Calcinosis** | **Current**  **Lipodystrophy** | **Current arthritis** |
| --- | --- | --- | --- | --- | --- | --- |
| ***Still taking medication (n=54)*** | | | | | | |
| Yes | 43 | 22 | 18 | 12 | 4 | 20 |
| No | 7 | 28 | 32 | 37 | 43 | 29 |
| Don’t know | 4 | 4 | 4 | 5 | 7 | 5 |
| ***Not taking medication (n=29)*** | | | | | | |
| Yes | 6 | 6 | 5 | 6 | 2 | 8 |
| No | 16 | 23 | 21 | 23 | 26 | 20 |
| Don’t know | 7 | 0 | 3 | 0 | 1 | 1 |
| ***p-*value**** | **< 0.001** | **0.033** | **0.274** | **0.222** | **0.376** | **0.380** |

*Responses from n=83

**From Chi-squared tests

**Supplementary Table S2:  Myositis-Specific and Myositis-Associated antibody status of questionnaire responders**

| **Myositis Specific or Associated Autoantibody*** | **Numbers positive** | **Diagnosis (*n*)** |
| --- | --- | --- |
| Anti-TIF1γ | 10 | Definite JDM (9)  Probable JDM (1) |
| Anti-NXP2 | 10 | Definite JDM (6)  JDM with Systemic Lupus Erythematosus (2)  JDM with Mixed Connective Tissue Disease (1)  Mixed Connective Tissue Disease (1) |
| Mi2 | 7 | Definite JDM (5)  Probable JDM (1)  Mixed Connective Tissue Disease (1) |
| Anti-U1RNP | 6 | Definite JDM (4)  JDM with Systemic Sclerosis (1)  JDM with Mixed Connective Tissue Disease (1) |
| Anti-MDA5 | 3 | Definite JDM (3) |
| Anti-HGMCR | 2 | Definite JDM (2) |
| Anti-Jo-1 | 2 | Definite JDM (2) |
| Anti-SRP | 2 | Definite JDM (2) |
| Anti-Ro | 1 | Definite JDM (1) |
| Anti-Topo-isomerase | 1 | JDM with Systemic Sclerosis (1) |
| Anti-SAE | 1 | Definite JDM (1) |
| Anti-Mi2 and Anti-NXP2 | 1 | Probable JDM (1) |
| Unknown Bands | 11 | Definite JDM (8)  Probable JDM (1)  JDM with Systemic Sclerosis (1)  JDM with Mixed Connective Tissue Disease (1) |
| Negative | 15 | Definite JDM (8)  Probable JDM (2)  JDM with Systemic Sclerosis (1)  JDM with Mixed Connective Tissue disease (1)  Mixed Connective Tissue disease (1)  Polymyositis with Systemic Sclerosis (1)  Other Idiopathic inflammatory Myopathy (1) |

*n = 73

**Supplementary Table S3**

**Responses to questions regarding growth and development**

| **Questionnaire parameter (*n*)** | **Yes**  **(n, %)** | **No**  **(n, %)** | **Don’t know**  **(n, %)** |
| --- | --- | --- | --- |
| **Feel shorter than peers (80)** | 20 (25.0) | 58 (72.5) | 2 (2.5) |
| **Feel weight was affected by myositis (80)** | 25 (31.3) | 43 (53.7) | 12 (15.0) |
| **Ever treated with growth hormone (79)** | 1 (1.3) | 77 (97.4) | 1 (1.3) |
| **Puberty affected by myositis (80)** | 16 (20) | 58 (72.5) | 6 (7.5) |
| **If puberty affected, have now caught up with peers (16)** | 12 (75) | 0 | 4 (25) |

**Supplementary Table S4: Height, weight and BMI percentiles at diagnosis and at time of questionnaire**

|  | **At Diagnosis** | **At time of**  **questionnaire** | ***p***  ***(n* = 50 paired data*)*** |
| --- | --- | --- | --- |
| **Mean Height**  **percentile (SD)** | 48.7 (15.2) | 52.0 (27.4) | 0.51 |
| **Mean Weight**  **percentile (SD)** | 56.8 (28.9) | 54.4 (29.0) | 0.89 |
| **Mean BMI**  **percentile (SD)** | 62.7 (29.8) | 23.6 (5.1) | 0.0136 |

**Supplementary Table S5: Questionnaire data regarding smoking compared to UK**

**ONS data**

| **Ever Smoked (*n*, %)** | **Currently Smoking (*n*, %)** | **UK ONS Data* %** |
| --- | --- | --- |
| 14/83 (16.9) | 2/83 (2.4) | 18.3 |

* Data were presented in ONS statistics as percentages only

**Supplementary Table S6: Alcohol intake of participants compared to UK ONS data**

| **Participants who have ever drunk alcohol**  ***n* (%)** | **Participants who currently drink alcohol , *n* (%)** | **2014 ONS data; those who currently drink alcohol in UK**  **(*n*, %)** | **Participants drinking more than 8 Units/week (males), or more than 6 units per week (females)**  ***n* (%)** | **2014 ONS data : those drinking more than 8 Units/week (males), or more than 6 units per week(females)**  ***n* (%)** |
| --- | --- | --- | --- | --- |
| 71/83 (85.5%) | 59/83 (71.1%) | 37.38 x10^6^/49.83 x10^6^ (74.9%) | 8/59 (13.6%) | 7.97 x10^6^/49.83 x10^6^ (16.0%) |

**Supplementary Table S7: Numbers with ≥ 3** **longitudinal values**

**for CMAS, MMT8, modified skin DAS and Physician Global VAS**

|  | **Questionnaire**  **Responders**  **(*n* = 84)** | **Questionnaire**  **sent but not returned (*p-***  **value)**  **(*n* = 106)** |
| --- | --- | --- |
| **CMAS**  Numbers with ≥ three  Longitudinal values  Median intensity index | 75  15.20 | 51 (*p* < 0.001)  7.37 |
| **MMT8**  Numbers with ≥ three  Longitudinal values  Median intensity index | 66  25.70 | 60 (*p* = 0.015)  13.50 |
| **Modified Skin DAS**  Numbers with ≥ three  longitudinal values  Median intensity index | 79  0.29 | 79 (*p* < 0.01)  0.20 |
| **Physician Global**  **assessment (VAS)**  Numbers with ≥ three  Longitudinal values  Median intensity index | 71  0.25 | 71 (*p* = 0.01)  0.30 |
